# Updates on Information Theory and Network Coding

**DOI:** 10.3390/e27010017

**Published:** 2024-12-30

**Authors:** Shenghao Yang, Kenneth W. Shum

**Affiliations:** School of Science and Engineering, The Chinese University of Hong Kong, Shenzhen 518172, China; wkshum@cuhk.edu.cn

Around the year 2000, network coding introduced the concept that coding can replace the basic packet forwarding operation used in traditional network communication systems [1,2,3]. This innovation simplified the achievement of maximum communication rates for unicast and improved the maximum communication rates for multicast. Random linear network coding (RLNC) bridged the gap between theory and practical applications by demonstrating that randomly selected linear combination coefficients are highly likely to be effective for linear network coding under some conditions [4,5]. Over the past two decades, significant research has focused on efficient RLNC. One notable approach is batched network coding (BNC), which integrates erasure coding with network coding in an outer-code inner-code manner (e.g., [6,7]).

Recently, network coding techniques have been discussed in 3GPP for 5G and within the Internet Research Task Force (IRTF), the research counterpart of the Internet Engineering Task Force (IETF). The Coding for Efficient Network Communication Research Group (NWCRG) of IRTF published six RFCs from 2018 to 2023. Network coding applications have been explored for satellite systems [8] and content-centric networking [9]. Two network coding protocols are described in [10,11], while the relationship between coding and congestion control is examined in [12].

As Guest Editors of this Special Issue of *Entropy*, titled “*Information Theory and Network Coding II*”, we are delighted to present ten cutting-edge research papers that explore advancements in coding theory and network coding. These papers cover a diverse array of subjects, such as finite-blocklength analysis (contribution 10), erasure coding (contribution 1), coded caching (contribution 8), vector-linear network coding (contribution 9), and secure network coding (contribution 6). Contribution 2 proposes a scalable RLNC scheme that adapts to the computational power of devices, while contribution 7 focuses on the application of RLNC in Vehicle-to-Vehicle (V2V) networks. Contributions 5 and 4 discuss the systematic design and adaptive recoding of BNC, respectively. Contribution 3 applies RLNC and BNC to distributed computation over lossy wireless networks.

We have witnessed rapid advancement in Large Language Models (LLMs) in recent years. This trend has provided numerous application scenarios for network coding and highlighted several promising directions for future research. Notably, training LLMs necessitates innovative GPU cluster networking technologies and distributed computation techniques. We eagerly anticipate the emergence of more groundbreaking research in network coding in the coming years.

We would like to express our sincere gratitude to all the authors who have contributed their high-quality work to this Special Issue. Their dedication and effort have made this collection of papers a reality. We also thank the reviewers for their time and expertise in evaluating the submitted manuscripts. Their constructive feedback has significantly enhanced the quality of the accepted papers. Furthermore, we are grateful to the Editorial Office of *Entropy* for their invaluable support and assistance in bringing this Special Issue to fruition.
